# Genome-wide identification and characterization of the *Lateral Organ Boundaries Domain* (*LBD*) gene family in polyploid wheat and related species

**DOI:** 10.7717/peerj.11811

**Published:** 2021-08-11

**Authors:** Jun Xu, Ping Hu, Ye Tao, Puwen Song, Huanting Gao, Yuanyuan Guan

**Affiliations:** 1 Henan Institute of Science and Technology, Xinxiang, China; 2 Henan Engineering Research Center of Crop/ Henan International Joint Laboratory of Plant Genetic Improvement and Soil Remediation Genome Editing, Xinxiang, China

**Keywords:** Triticeae species, Genome-wide analysis, LBD gene family, Collinearity, Evolutionary history of homologs

## Abstract

**Background:**

Wheat (*Triticum aestivum*) originated from three different diploid ancestral grass species and experienced two rounds of polyploidization. Exploring how certain wheat gene subfamilies have expanded during the evolutionary process is of great importance. The *Lateral Organ Boundaries Domain* (*LBD*) gene family encodes plant-specific transcription factors that share a highly conserved LOB domain and are prime candidates for this, as they are involved in plant growth, development, secondary metabolism and stress in various species.

**Methods:**

Using a genome-wide analysis of high-quality polyploid wheat and related species genome sequences, a total of 228 *LBD* members from five Triticeae species were identified, and phylogenetic relationship analysis of *LBD* members classified them into two main classes (classes I and II) and seven subgroups (classes I a–e, II a and II b).

**Results:**

The gene structure and motif composition analyses revealed that genes that had a closer phylogenetic relationship in the same subgroup also had a similar gene structure. Macrocollinearity and microcollinearity analyses of Triticeae species suggested that some *LBD* genes from wheat produced gene pairs across subgenomes of chromosomes 4A and 5A and that the complex evolutionary history of *TaLBD4B-9* homologs was a combined result of chromosome translocation, polyploidization, gene loss and duplication events. Public RNA-seq data were used to analyze the expression patterns of wheat *LBD* genes in various tissues, different developmental stages and following abiotic and biotic stresses. Furthermore, qRT-PCR results suggested that some *TaLBDs* in class II responded to powdery mildew, regulated reproductive growth and were involved in embryo sac development in common wheat.

## Introduction

*Triticum aestivum* is a global staple crop of the Poaceae family, providing large amounts of energy and protein for human diets (Shiferaw et al., 2013; Veraverbeke & Delcour, 2002). Today’s bread wheat originated from three different diploid ancestral grass species and resulted from two consecutive hybridizations: about 500,000 years ago, a tetraploid ancestor of today’s durum wheat *T. turgidum* (AABB) resulted from a combination of *T. urartu* (A-genome donor) and a relative of today’s *Aegilops speltoides* (B-genome donor); and about 8000 years ago, after the second hybridization of *Ae. tauschii* (D-genome donor) and *T. turgidum*, the hexaploid ancestor (AABBDD) of today’s bread wheat was formed (Allaby et al., 2017; Shewry, 2009). Because of the large genome (about 16 Gbp) and two rounds of polyploidization of hexaploid wheat, the genetics, functional genomics and breeding programs have been a challenge (Borrill, Adamski & Uauy, 2015). However, rapid development of high-throughput sequencing technologies has led to a high-quality genome assembly and annotation of common wheat (*Triticum aestivum*) and its relatives (IWGSC, 2018; Avni et al., 2017; Clavijo et al., 2017; Ling et al., 2018; Ling et al., 2013; Luo et al., 2017; Mascher et al., 2017; Zimin et al., 2017). Furthermore, large-scale RNA-seq analyses of wheat genes at different developmental stages and under a variety of stresses have provided convenient conditions for more detailed analyses (Ramirez-Gonzalez et al., 2018).

Genes of the *Lateral Organ Boundaries* (*LOB*) *Domain* (*LBD*) encode plant-specific transcription factors that share a highly conserved LOB domain, and are also known as the *ASYMMETRIC LEAVES2-like* (*AS2*) gene family (Iwakawa et al., 2002). The first *LBD* gene was identified in *Arabidopsis* based on the special gene expression pattern of an enhancer trap insertion, which is expressed in a band of cells at the adaxial base of all lateral organs (Shuai, Reynaga-Pena & Springer, 2002). The *LBD* family can be classified into two classes, classes I and II, according to the specific protein sequences of the LOB domain in the N-terminus; class I have a conserved CX2CX6CX3C zinc finger-like motif with potential DNA-binding ability, a GAS (Gly-Ala-Ser) sequence, which may also play a role in DNA-binding; and an LX6LX3LX6L leucine zipper-like coiled-coil motif, which presumably participates in protein dimerization. In contrast, class II only contain a conserved zinc finger-like motif *(*Iwakawa et al., 2002; Lu et al., 2018; Shuai, Reynaga-Pena & Springer, 2002). The variable C-terminal region of the LBD proteins plays a critical role in controlling the expression of downstream target genes (Liu et al., 2005; Majer et al., 2012). The *LBD* genes are found only in plants, implying that this gene family may regulate plant-specific growth and development processes (Shuai, Reynaga-Pena & Springer, 2002). Previous research of *LBD* type genes in various plant species indicated that *LBD* family genes have various functions and are involved in many aspects of plant organ or tissue growth and development, including lateral roots, stems, leaves, embryo sacs, inflorescences and flowers (Liu et al., 2005; Xu et al., 2008; Xu, Luo & Hochholdinger, 2016; Xu et al., 2003). The *LBD* genes are also involved in anthocyanin accumulation, nitrogen metabolism and pathogen response. (Bortiri et al., 2006; Lu et al., 2018). The *LBD* type genes have been phylogenetically and functionally characterized in *Arabidopsis* and a variety of important crop plants, such as *Hordeum vulgare*, *Oryza sativa*, *Setaria italica*, *Sorghum bicolor*, *Zea mays*, *Glycine max* and *Gossypium raimondii*, which each have 28–131 members (Zhang et al., 2020).

Previous studies confirmed that LBD proteins play essential roles in the regulation of the growth and development of varieties of plants. Two *LBD* genes in *Arabidopsis*, *AS1* and *AS2* genes can form symmetrical leaves by inhibiting *Knox* gene expression (Phelps-Durr et al., 2005). Genes *AtLBD16*, *AtLBD18* and *AtLBD29* are involved in lateral root initiation, and *AtLBD29* is involved in auxin signaling that regulates fiber wall biosynthesis (Goh et al., 2019; Lee et al., 2019a; Lee et al., 2019b). The wheat *LBD* gene *TaMOR* can improve root architecture and increase yield in crop plants (Li et al., 2016). In *Arabidopsis*, AtLBD10 interacts with AtLBD27 to control pollen development (Kim et al., 2015). In rice, *OsIG1* is involved in the development of floral organs and the megagametophyte (Zhang et al., 2015). A maize ortholog of LOB, *RA2*, has been shown to regulate reproductive growth and is involved in morphogenesis of the maize inflorescence (Bortiri et al., 2006; Vollbrecht et al., 2005). Some *LBD* genes are involved in metabolism: *AtLBD37*, *AtLBD38* and *AtLBD39* from class II repress anthocyanin biosynthesis and regulate nitrogen metabolism (Rubin et al., 2009); *EgLBD37* leads to significantly increased secondary xylem, and *EgLBD29* impacts phloem fiber production of *Eucalyptus grandis* (Lu et al., 2018). Additionally, the MdLBD13 protein can affect anthocyanin synthesis and nitrogen uptake in apple (Li et al., 2017).

Some studies also reported that *LBD* is involved in response to different biotic and abiotic stresses. Members of these *LBD* gene families regulate gene expression in response to a range of biotic stimuli, including microbes (bacteria, fungi and oomycetes) and insects (Eulgem, 2005; Thatcher, Kazan & Manners, 2012a). Silencing of *AtLBD20*, a *Fusarium oxysporum* susceptibility gene, results in increased resistance to the pathogen (Thatcher et al., 2012b); *CsLOB1* is a citrus bacterial canker susceptibility gene in C*itrus sinensis* (Hu et al., 2014; Zhang et al., 2017); *AtLBD16* provides a molecular link between lateral roots and root-knot nematode feeding sites during the interaction of *Arabidopsis–Meloidogyne* spp (Cabrera et al., 2014). In potato, expression of *StLBD2-6* and *StLBD3-5* in the stem was induced under drought stress (Liu et al., 2019); in soybean, *GmLBD12* responded to drought, salt, cold, indole acetic acid, abscisic acid and salicylic acid treatments (Yang et al., 2017). Expression profiles of the *LBD* genes in *Arabidopsis* that encode the smaller class-II LBD proteins were responsive to multiple pathogens, suggesting functions in plant defense responses (Thatcher, Kazan & Manners, 2012a). Thus, understanding *LBD* genes is important for understanding plant development, which is in turn crucial for plant breeding and crop improvement.

Although overviews of *LBD* family members have been identified and functionally characterized in several plant species, a detailed genome-wide phylogenetic and functional characterization of wheat and related species *LBD* genes is still missing. The genomes of some wheat ancestors have been sequenced, which makes genome-wide identification of a gene family in Triticeae species feasible. An important goal of future research projects will be to infer when expansion of certain wheat gene subfamilies has occurred during the evolution process (Schilling et al., 2020). To better understand the dynamics of *LBD* gene evolution in Triticeae species and to facilitate future research on this important gene family, we used bioinformatic methods to identify *LBD* genes in polyploid wheat and related species. The classification, structure, conserved motifs, macrocollinearity, microcollinearity among species, functional diversification and expression pattern of these genes in various tissues, different development stages and in response to different stresses were systematically analyzed. This work will help us to understand the evolution and diversification of the *LBD* genes among Triticeae species and their potential roles in plant growth and responses to stresses, and provide a foundation for future functional studies of these genes.

## Materials & Methods

### Plant materials and plant growth conditions

The common wheat cultivar Bainong207 from Henan Institute of Science and Technology, was used for gene expression analysis. Tissue expression of *TaLBDs* in root, stem and leaf were examined at the seedling stage and embryo sac, lodicule, glume, glumelle and lemma were collected at bicellulate pollen stage for quantitative real-time reverse transcription polymerase chain reaction (qRT-PCR) analysis, the materials was grown under natural conditions in the field at Huixian Experimental Station, Henan Institute of Science and Technology.

### *Blumeria graminis* f. sp. *Tritici* (*Bgt*) races preparation and plant treatments

Mixed races of *Bgt* were collected in the field of Xinxiang experimental station (Xinxiang, China) and preserved on seedlings of the high susceptible variety Sumai3 in the greenhouse. The seedling of Bainong207 was used for gene expression analysis. For RNA extraction, *Bgt* inoculation was performed by spraying fresh spores from Sumai3 to the seedling leaves, and leaves of Bainong207 (susceptiable to mixed races of *Bgt*) was inoculated with mixed races and the leaf tissues were sampled at 0, 12, 24 and 36 h after inoculation. For the abiotic stress treatment, 14 day-old wheat seedlings was treated with 200 mM NaCl and leaves were collected after 12 and 24 h. All these two materials of Bainong207 and Sumai3 were grown in a greenhouse under a 14 h light/10 h dark cycle at 22 °C /18 °C, with 70% relative humidity.

### Expression analysis of *TaLBDs* by qRT-PCR

The methods for total RNA isolate, the first-strand cDNA synthesis, qRT-PCR program and the relative values of gene expression were performed as previously described in Hu et al. (2018). Primers used for expression analysis of *TaLBDs* by qRT-PCR were listed in Table S1. SigmaPlot 14 was used to develop figures.

### Identification of *LBD* gene families

All the Genome-wide data for *Triticum aestivum L.* (Chinese Spring wheat cultivar) wheat from IWGSC (http://www.wheatgenome.org/) and The Genome Analysis Centre (TGAC) (https://opendata.earlham.ac.uk/opendata/data/Triticum_aestivum/TGAC/v1/annotation/) were downloaded (IWGSC, 2018; Clavijo et al., 2017). Data for *Triticum urartu* (Tu 2.0) were downloaded from the MBKBase website (http://www.mbkbase.org/Tu/) (Ling et al., 2018). *Triticum dicoccoides* (WEWSeq_v.1.0), *Aegilops tauschii* (Aet_v4.0), *Hordeum vulgare* (IBSC_v2), https://plants.ensembl.org/Oryza_indica/Info/Index?db=core) and *Brachypodium distachyon* (*Brachypodium_distachyon_v3.0*) were downloaded from the Ensemble Plants website to construct a local database (http://plants.ensembl.org/index.html). The typical LOB domains (PF03195) were downloaded from the Pfam database as the search models (http://pfam.xfam.org/) (El-Gebali et al., 2019). To ensure the search results was reliable, a new Hidden Markov Model (HMM) was built. From the proteins obtained using the raw LOB HMM, a high-quality protein set (*E*-value <1 × 10^−20^ and manual verification of an intact LOB domain) was aligned and used to construct a specific LOB HMM using hmmbuild from the HMMER v3 suite (Lozano et al., 2015*)*. This new specific HMM was used, and all proteins with an *E*-value lower than 0.001 were selected. If a gene has multiple transcripts, only the longest one was retained for the subsequent analysis.Comparing the LBD sequences searched from two common wheat database IWGSC and TGAC, the repetitive sequences were eliminate and each of the corresponding sequences just preserve one. All candidate LBD protein sequences were examined by the domain analysis programs SMART (Simple Modular Architecture Research Tool) (http://smart.embl-heidelberg.de/) (Letunic, Doerks & Bork, 2015) and Conserved Domains (https://www.ncbi.nlm.nih.gov/Structure/cdd/wrpsb.cgi) (Lu et al., 2020). All the proteins have a complete LOB domain except *AET5Gv20661300.1* (*AetLBD-5D*), *AET4Gv20808800.1* (*AetLBD-4D*) from *Ae. tauschii* were retained.

### Naming of *LBD* genes in Triticeae species

Each gene name starts with an abbreviation for the species name *T. aestivum* (Ta), *T. dicoccoides* (Td)*, Ae. tauschii* (Aet), *T. urartu* (Tu). Taking into their subgenome location (A, B or D), the gene names include an A, B or D to indicate the subgenome they were located, for example *TaLBD2A-1*, indicated that this gene was from the 2A subgenome of common wheat. Genes belonging to one chromosome were consecutively numbered (e.g., *TaLBD4D-1* to *TaLBD4D-11*), all gene names are listed in Table S2.

### Phylogenetic, gene structure and motif composition analysis

Multiple sequence alignment of all these LBD proteins (including two incomplete LBD sequence from *Ae. tauschii*) were performed with ClustalW using the default options in MEGA-X (Kumar et al., 2018). Phylogenetic trees were constructed based on bootstrap neighbor-joining (NJ) algorithms with the Kimura two-parameter model of MEGAX (Grimplet et al., 2017; Liu et al., 2019; Lu et al., 2018; Rui-Min et al. 2018; Yu et al., 2020a; Yu et al., 2020b). The stability of the internal nodes was measured by bootstrap analysis of 1,000 replicates (Yu et al., 2020a; Yu et al., 2020b). The illusion of “Long-branch Attraction” (LBA) exists widely in molecular phylogenetic analysis. The most commonly used methods, such as distance based methods represented by NJ and character based methods represented by maximum likelihood method all have this disturbing factor (*Bergsten, 2005*).  All methods will inevitably appear “LBA”, but some measures were taken to reduce the occurrence of this phenomenon in the process of phylogenetic analysis. Firstly of all, NJ  has the characteristics of high speed and high efficiency, which is applicable for evolutionary studies in close entities (*Godini & Fallahi, 2019*). In the present study, most of the analyzed taxa were closely related species of Triticeae, it is accorded with the content of the method  “breaking long branch method” to reduce the problem of “LBA”, in other words, taxonomic elements with close relationship were added for phylogenetic analysis. Secondly, in order to avoid the great difference among species, and refered to the method described by Zhao et al. (2019), the conseved LOB domain were used for construct the evolutionary tree, which would further reduces the possibility of  “LBA” (*Bergsten, 2005; Zhao et al., 2019*). The phylogenetic tree was visualized with EvolView (https://www.evolgenius.info/) (He et al., 2016).

The gff3 files of each species was downloaded from the Ensemble Plants FTP server (http://plants.ensembl.org/index.html) for exon–intron structure analysis, gene structures were generated using TBtools (Chen et al., 2020a), based on the full-length genome sequence and the corresponding coding sequences of *LBD* genes. Motif analysis was performed using the MEME program (http://memesuite.org/tools/meme) the parameters were employed as the following descriptions: the maximum number of motifs, 20; and the optimum width of each motif, between 6 and 50 residues *(*Xie et al., 2018). The characteristics of *LBD* gene structure with motif composition were visualized by the TBtools.

### Chromosome localization, gene duplication and synteny analysis

The Multiple Collinearity Scan toolkit (MCScanX) was used to determine *LBD* gene duplication, synteny and collinearity related to other representative species (Wang et al., 2012). The shinyCircos software (http://shinycircos.ncpgr.cn) was applied to express the syntenic relationship of the gene pairs and their respective loci in the wheat genome (Yu, Ouyang & Yao, 2018).

Microcollinearity is valuable to understand the investigation of gene loss during evolution or the evolution of specific genes in a local regions. TGT (Triticeae-Gene Tribe, http://wheat.cau.edu.cn/TGT/) was used to trace the origin history of the target gene, and gene pair was also analyzed in this website as described in Chen et al., (2020b).

### Functional diversification analysis

The analysis of functional diversification among the subgroups was performed using DIVERGE v3.0 software based on the selected protein sequences. The coefficients of functional diversification theta of Type I (MFE Theta) and Type II (Theta-II) were calculated, as a measure of functional differentiation, theta fluctuates between 0 and 1. If the difference between theta and 0 was significant, it means that there was significant functional differentiation. Type-I functional differentiation is defined as functional differentiation caused by the change of evolution rate, Type II functional differentiation is defined as the evolution rate does not change, but the characteristics of amino acids changes resulting in functional differentiation (Gu et al., 2013).

### RNA-seq Expression Analysis

RNA-seq data of 93 (*TaLBD4B-2* was deficient) wheat *LBD* genes were downloaded from the expVIP (http://www.wheat-expression.com/) (Borrill, Ramirez-Gonzalez & Uauy, 2016). The tissue-specific expression data of three wheat tissues (root, stem, leaf) and spikes were collected from Chinese Spring at seedling and anthesis stages, respectively. Developmental stages refer to seedling, vegetative and reproductive stages from Chinese Spring wheat. The biotic stress of powdery mildew and abiotic stress expression data (heat and drought) was collected from N9134 (powdery mildew-resistant wheat at seedling stage) and TAM107 (heat-resistant wheat at seeding stage), respectively. The relative expression of each *TaLBD* gene in different tissues, developmental stages and stresses were presented as a heat map, which was constructed by TBtools (Chen et al., 2020a). Moreover, we used qRT-PCR to study the gene expression patterns in various tissues and the transcriptional responses of some *TaLBDs* to powdery mildew. The sequences of gene-specific primers are shown in Table S1.

## Results

### Identification and phylogenetic relationship analysis of the *LBD* gene family in Triticeae

**Figure 1:**
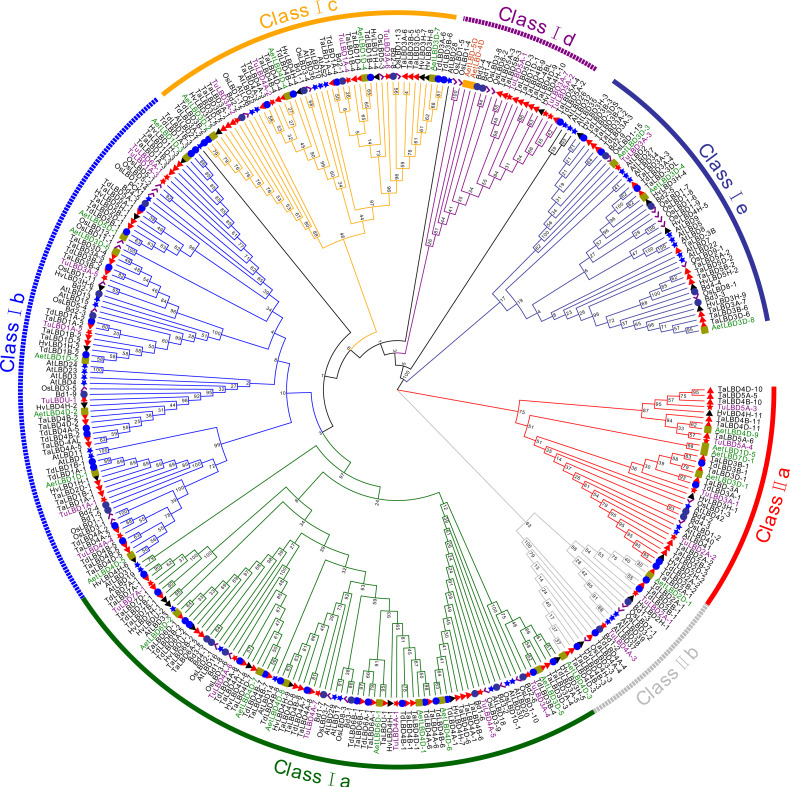
Phylogenetic relationship analysis of 338 LBD proteins from *Arabidopsis*, rice, *B. distachyon*, *H. vulgare*, *T. urartu*,* Ae. tauschii*, *T. dicoccoides* and *T. aestivum*. The phylogenetic tree was built using the neighbor-joining method with 1000 bootstrap replicates by MEGA X. The diverse subgroups of LBD proteins are marked with different colors. The LBD proteins of *Arabidopsis*, rice, *B. distachyon*, *H. vulgare*, *T. urartu*, *Ae. tauschii*, *T. dicoccoides* and *T. aestivum* are represented by blue stars, purple checkmarks, purple circles, black triangles, red stars, green squares (except for two incomplete LBDs from *Ae. tauschii* indicated by orange squares), blue circles and red triangles, respectively. Gene IDs of all analyzed genes can be found in Table S2.

In total, 228 *LBD* genes with a complete LOB domain were identified from the public databases of five analyzed Triticeae species. There were 27, 27, 49, 31 and 94 (88 from IWGSC and six from TGAC databases) *LBD* genes in *T. urartu*, *Ae. tauschii*, *T. dicoccoides*, *H. vulgare* and common wheat, respectively. The evolutionary relationship of 230 Triticeae *LBD*s (including two from *Ae. tauschii* which did not have a complete LOB domain), along with 28 from *B. distachyon*, 37 from rice and 43 from *Arabidopsis* were phylogenetically analyzed (Fig. 1, Table S2). According to a phylogenetic tree and branch lengths, these *LBD* genes were divided into two major classes, classes I and II; class I was further assigned to five subgroups, classes I a–e; and class II was further assigned to two subgroups, classes II a and II b (Fig. 1). In total, there were 274 (81.55%) and 57 (16.36%) *LBD* genes with a complete LOB domain in classes I and II of the above species, respectively (Fig. 1, Table 1). Therefore, the number of *LBD* genes in class I was approximately five times that in class II of analyzed species. Subgroup class I d was the smallest in class I with 19 *LBD* genes, and subgroup class I a was the largest with 85 (Table 1). In most cases, the *LBD* members of Triticeae species were clustered together in the same subgroup, and the *LBD* members of *Arabidopsis* were clustered together, indicating that *LBD* genes of monocotyledons and dicotyledons were greatly differentiated during the evolutionary process. The similarity of the protein sequences in the same subgroup from the Triticeae species was high, indicating the evolutionary process of the genes in the same subgroup may have been relatively conservative. Based on the tree topology and branch lengths, five *Arabidopsis LBD* genes (*AtLBD21*, *AtLBD28*, *AtLBD26*, *AtLBD32* and *AtLBD35*) were not classified into the seven subgroups (Fig. 1). *Aegilops tauschii* was the D-genome donor of common wheat, however, there was no LBD protein in class I d. Interestingly, when two *LBD* genes from *Ae. tauschii* that did not have a complete LOB domain (*AetLBD-4D* and *AetLBD-5D*) were retained to construct phylogenetic trees, they were clustered into class I d. Therefore, *AetLBD-4D* and *AetLBD-5D*, which did not contain a complete LOB domain, were retained in the following analysis.

**Table 1:** Numbers of *LBD* homologous encoded by the surveyed genomes in total and individual subgroups.

Genome	Total number	Subgroup I					Subgroup II		Other
		a	b	c	d	e	a	b	
*H. vulgare* (HH)	31	8	7	5	1	5	3	2	
*T. urartu* (AA)	27	6	7	4	3	1	4	2	
*Ae. tauschii* (DD)	27+*2*	7	7	4	*2*	3	4	2	
*T. dicoccoides* (AABB)	26 (49)	8 (16)	7 (13)	4 (8)	1	2 (3)	2 (4)	2(4)	
*T. aestivum* (AABBDD)	36 (94)	8 (23)	7 (22)	4 (12)	3 (8)	8 (12)	4 (12)	2(5)	
*B.distachyon*	28	8	6	4	2	4	2	2	
*Oryza sativa*	37	7	10	4	4	7	2	3	
*Arabidopsis*	43	10	9	5	0	8	3	3	5
Total	336+*2*	85	81	46	19+*2*	43	34	23	5

**Notes.**

Numbers in brackets indicate number of copies for polyploid genomes.

Numbers in italics represent two incomplete *LBD* genes from *Ae. tauschii*.

### The number of *LBDs* in genomes of Triticeae

The subgroups comprising members of all the analyzed Triticeae species are listed in Table 1. Except for *Arabidopsis*, *LBD* genes from the different analyzed species were distributed in each subgroup. In class II b, each of the five analyzed Triticeae species, *H. vulgare*, *T. uratu*, *Ae. tauschii*, *T. dicoccoides* and *T. aestivum*, had two *LBD*s*.* In class I b,  all the five analyzed Triticeae  species had seven *LBD*s. In class I c, *H. vulgare* had five *LBD*s and the other four species had four *LBDs* (Table 1). The number of *LBDs* from different Triticeae species in classes I a, I d, I e and II a significantly differed, possibly due to either the quality of the genome assembly or chromosome translocation, duplication or deletion during evolution. Compared with each subgroup of *Arabidopsis*, the numbers of *LBD* genes from *B. distachyon* and  rice were closer to those of wheat and its related species in the corresponding subgroups (Table 1), indicating that *LBD* genes experienced great evolution following the differentiation of monocotyledons and dicotyledons.

*T. dicoccoides* and *T. aestivum* had 49 and 94 *LBD*s, which was about two and three times of that in diploid species, respectively. Moreover, the number of *LBD*s in each subgroup was almost 2–3 times that of the diploid species, except for classes I d, I e and II a (Table 1). This indicated that the increased number of *TaLBDs* in polyploid wheat was mostly due to genome polyploidization. It is interesting that there was only one *LBD* gene of *T. dicoccoides* in class I d, compared to three in *T. uratu* and eight in *T. aestivum*; this may be due to either a poor reference genome sequence of *T. dicoccoides* or gene loss events during *T. dicoccoides* evolution*.*

### Chromosome distribution and gene duplication of *TaLBD* genes

The identified 228 *LBD*s from five Triticeae species (*T. aestivum*, *Ae. tauschii*, *T. uratu*, *T. dicoccoides* and *H. vulgare*) were assigned to corresponding chromosomes (four had unknown chromosomes). The number of *LBD*s in each subgenome of the same species was almost the same; with the exception of *H. vulgare* chromosome 6H, all chromosomes had at least one *LBD* gene (Table 2). In *T. aestivum*, 32, 31 and 30 *LBDs* were identified in the A, B and D subgenomes, respectively. In *T. dicoccoides*, 25 and 24 *LBDs* were identified in A and B subgenomes, respectively. In *Ae. tauschii*, *T. uratu* and *H. vulgare*, 27, 29 and 31 *LBDs* were identified. Compared with *T. dicoccoides*, there were seven more *LBDs* of *T. aestivum* in subgenomes A and B (Table 2). The *LBDs* from the five Triticeae species were not evenly distributed among chromosomes or different homologous groups. The numbers of *LBDs* in homologous groups 1, 2, 6 and 7 of each subgenome were similar, while the number of *LBD*s in homologous groups 3, 4 and 5 of each subgenome significantly differed (Table 2). In homologous groups 1 and 2, the number of *LBD*s in all subgenomes of Triticeae species was four and two, except for *Ae. tauschii* with five and one, respectively. In total, there were 33, 15, 59, 75, 22, 12 and 8 *LBDs* in the homologous groups 1–7, respectively; group 4 had the most *LBD*s (75, 32.89%), followed by group 3 (59, 25.88%) and group 7 had the least (8, 3.51%) (Table 2). The numbers of *LBD*s on chromosome 5A of *T. aestivum* (six) and *T. dicoccoides* (two) were twice those of chromosome 5B in the corresponding species, and the number of *LBD*s on *T. uratu* chromosome 5A (four) was higher than on chromosome 5A of *T. dicoccoides* (two) (Table 2). At the same time, the *LBD*s on chromosome 4A of *T. uratu* were less than other diploid Triticeae species, so we focused on the *LBD* evolution on chromosomes 4 and 5.

**Table 2:** Number of *LBD* from different species in each of the chromosomes.

Chromosome	*T. aestivum*			*T. dicoccoides*		*T.urartu*	*Ae. tauschii*	*H. vulgare*	Total
	A	B	D	A	B	A	D	H	
Chr.1	4	4	4	4	4	4	5	4	33
Chr.2	2	2	2	2	2	2	1	2	15
Chr.3	7+*1*	6+*1*	6+*2*	6	6	7	8	9	59
Chr.4	8+*1*	11+*1*	11	8	8	7	9	11	75
Chr.5	6	3	3	2	1	4	1	2	22
Chr.6	2	2	1	2	2	1	2	0	12
Chr.7	1	1	1	1	1	1	1	1	8
Total	32	31	30	25	24	26	27	29	224
^*^Unknow	1					1		2	4

**Notes.**

* The genes that were assigned to unknown chromosome. The italics represent *LBD* genes found in TGAC v1database.

**Figure 2:**
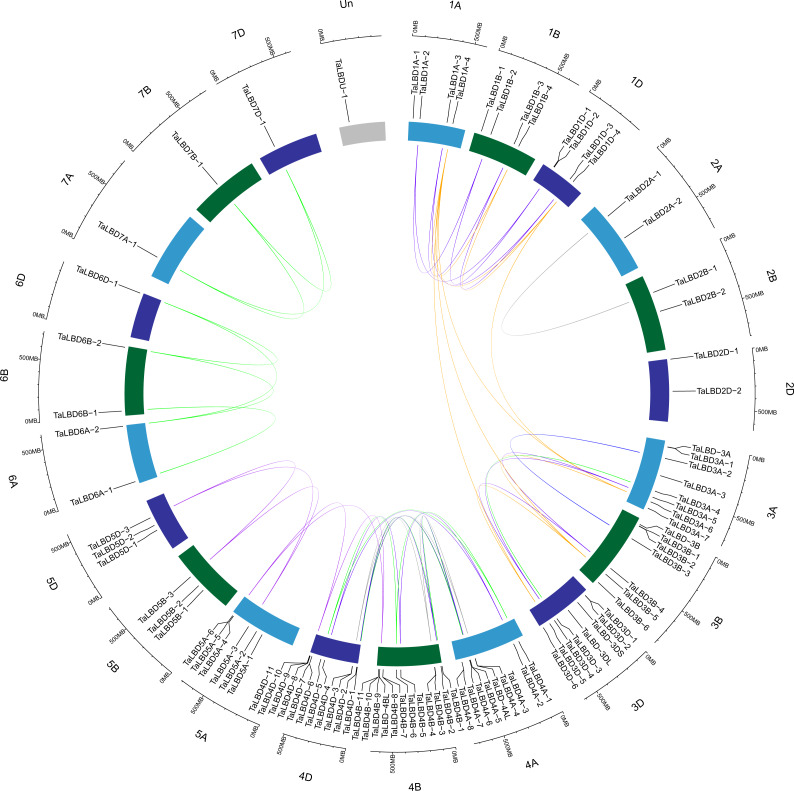
Homologous gene pairs and location of *LBD* genes of wheat. All *LBD* genes were mapped to their respective loci in the wheat genome in a circular diagram using shinyCircos. Subgenomes A, B and D are indicated by light blue, dark green and dark blue, respectively. Homologous genes were inferred by TGT (for details see Materials and Methods) and linked with specific colors.

In previous reports, segmental and tandem duplications have been suggested as two of the main causes of gene family expansion during the evolution of genomes and genetic systems in plants (Cannon et al., 2004; Moore & Purugganan, 2003, Yu et al., 2020a; Yu et al., 2020b). Compared with *Arabidopsis*, rice, *B. distachyon*, *T. urartu*, *Ae. tauschii*, *T. dicoccoides* and *H. vulgare*, which contained 43, 37, 28, 27, 27 (29), 49 and 31 *LBD* genes, respectively, the common wheat *LBD* family was remarkably large at 94 members, suggesting that the expansion of *LBD*s in common wheat occurred more rapidly, possibly a result of gene duplication and two rounds of polyploidization (Chen et al., 2020b; Dubcovsky & Dvorak, 2007; Hao et al., 2020). Thus, we investigated the duplication patterns of the common wheat *LBD* family. In the present study, the genome synteny analysis was used to investigate the expansion mechanism of the *LBD* gene family and shinyCircos software was used to show the the syntenic relationship of the gene pairs and their respective loci in the wheat genome (Fig. 2).

There were more *LBDs* in homologous groups 3 and 4, with group 4 having the most *LBDs*, followed by group 3; the genes of group 3 were distributed evenly on the whole chromosome, but genes of group 4 were mostly distributed at both ends of the chromosome (Fig. 2). The MCScanX analysis showed no evidence of tandem duplication or segmental duplication. There were 50 pairs of *LBD*s in common wheat, and 44 (88%) of them occurred in the same homologous group; furthermore, other gene pairs were mostly located on homologous groups 4 and 5. This may be due to structural rearrangements of chromosomes 4A–5A–7B in common wheat in two major translocation events (Chen et al., 2020b; Dvorak et al., 2018; Jorgensen et al., 2017). The duplication of *LBDs* in *T. dicoccoides* was also analyzed, which showed only one tandem duplication event, *TdLBD4A-7* and *TdLBD4A-8*. In common wheat and *T. dicoccoides*, there were few or no tandem repeat or segmental duplication of *LBD* genes, indicating that tandem repeat and segmental duplication had no significant effect on evolution of *LBDs* in common wheat and *T. dicoccoides*.

### Gene structure and motif composition analysis

**Figure 3:**
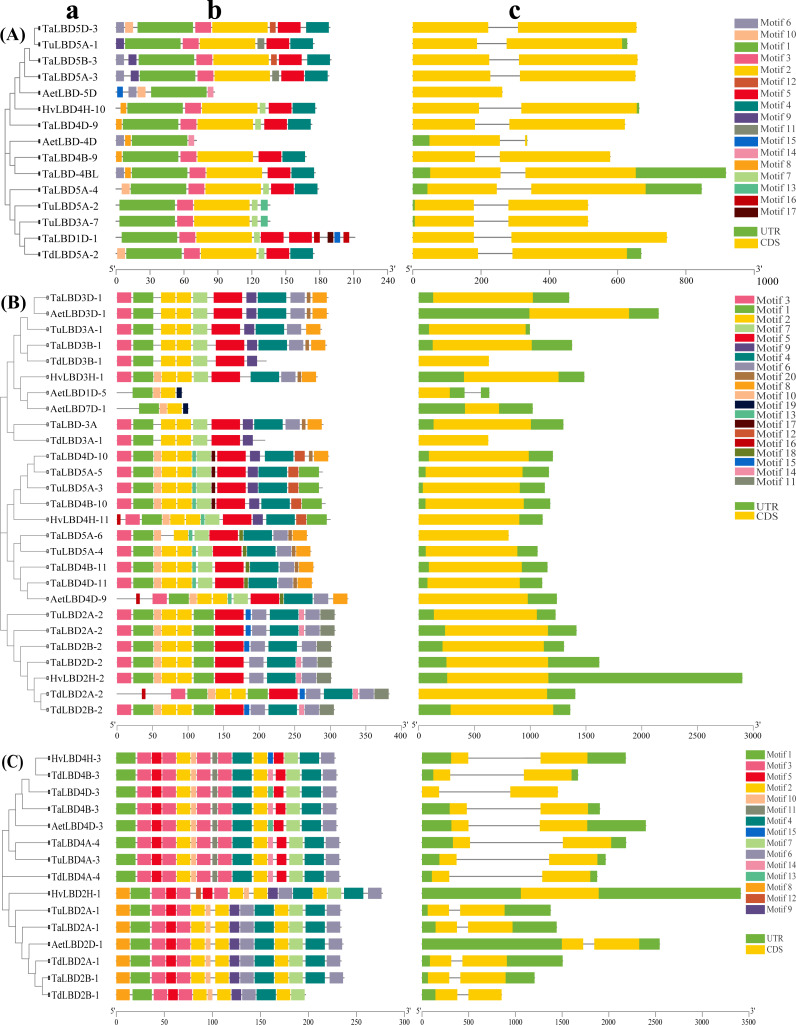
Phylogenetic relationships, gene structures and conserved protein motifs of *LBD* genes in Triticeae species. (A–C) Phylogenetic tree, protein motifs and gene structures of Triticeae species *LBD* s grouped into classes I d, II a and II b, respectively. (A) The phylogenetic tree was constructed using the neighbor-joining method with 1,000 bootstrap replicates by MEGA X. (B) The motif composition of LBD proteins. The motif compositions were analyzed by the online tool MEME, different motifs for LBD proteins are indicated by different colored boxes and numbered 1–20. (C) Exon–intron structure of *LBDs*. Gene structure analysis of *LBD* genes was performed using TBtools.

Exon–intron structure divergences play an important role during the evolution of duplicate genes and the structure of genes is important for determining their expression and function (Cao, 2019; Xu et al., 2012). To further explore the possible evolutionary relationship and the possible function of LBD proteins in Triticeae species, LBDs were analyzed based on phylogenetic trees, gene structure and conserved motifs. The alignment of full-length cDNA with genomic DNA sequence was used to analysis the intron–exon structure (Fig. 3, Fig. S1). Among all the analyzed 230 *LBD*s in the seven subgroups, 92 (40.00%) *LBD*s had no intron, with 16, 14, 16, 1, 17, 27 and 1 in the subgroup of classes I a to II b; and all the *LBDs* in class II a had no intron; 124 (53.91%) had one intron, and there were 44, 29, 17, 14, 6, 0 and 14 in the subgroup of classes I a to II b; 13 (5.65%) had two introns, and all the *LBD*s with two introns were in subfamily class I b and only one (0.43%) *LBD* gene had more than three introns (Fig. 3, Fig. S1). Along with the seven subgroups, classes I d and II b (except *HvLBD2H-1*) had only one intron and class II a had no intron (Fig. 3A-Fig. 3C), and may indicate that their biological roles differ from that of other subgroups. The genes had a closer phylogenetic relationship in the same subgroup, and also had a similar gene structure, indicating that the phylogenetic relationship among *LBD*s were highly correlated with exon–intron structure.

To further explore the possible evolutionary relationship of LBD proteins in Triticeae species, the motifs of the LBDs from different species were analyzed. Conserved motifs of these proteins were identified using MEME software. Here, 20 conserved motifs (named motifs 1–20) were identified, and the results shown in schematic diagrams (Fig. 3, Fig. S1). The motif composition of LBDs within the same subgroup, especially in the same species, were similar, indicating that the *LBD* gene family was evolutionarily conserved in the same subgroup and may have similar functions. The LBDs of Triticeae species possessed 2–18 motifs, and most of them contained both motifs 1 and 2 (Fig. 3, Fig. S1). Most of the motif structures of LBDs in Triticeae species were similar to their ancestors’ LBD protein; for example, in class II a, the motifs in TaLBD2A-2, TaLBD2B-2 and TaLBD2D-2 were similar to motifs in TuLBD2A-2, TdLBD2A-2 and TdLBD2B-2, correspondingly (Fig. 3B). In addition to containing motifs 1 and 2, homologs of *TaLBD3B-1*, *TaLBD3D-1* and *TaLBD3A* encoded proteins also containing motifs 3–6, 8, 9 and 20 (Fig. 3B); *TaLBD3A* was searched from the TGAC v1 database, and TGAC v1 is assembled different to IWGSC RefSeqv1.1, suggesting that protein sequences obtained from both databases were more comprehensive (Schilling et al., 2020). Interestingly, in class II a, the motifs of TaLBD4D-10 were exactly the same as for TaLBD5A-5 and TaLBD4B-10, and also similar to TuLBD5A-3, but different to the LBD protein in the same subgroup of *Ae. tauschii* (Fig. 3B); additionally, *TaLBD5A-5*, *TaLBD4B-10* and *TaLBD4D-10* did not belong to the same homologous group. The same situation also occurred for proteins TaLBD5A-6, TaLBD4B-11 and TaLBD4D-11 in class II a and TaLBD4B-9, TaLBD4D-9, TaLBD5A-4 in class I d (Figs. 3A–3B). The *TaLBD5A-3*, *TaLBD5B-3* and *TaLBD5D-3* were gene pairs, but the motifs of the three proteins were not identical; furthermore, TaLBD5A-3 was more similar to TuLBD5A-1, TaLBD5D-3 and AetLBD-5D, and they all contained motif 10 (Fig. 3A). In addition, the motifs of the gene pairs *TaLBD4B-9*, *TaLBD4D-9* and *TaLBD5A-4* were also not identical, and AetLBD-4D, HvLBD4H-10, TaLBD4D-9, TaLBD4B-9 and TaLBD-4BL specifically contained motif 8 (Fig. 3A).

### Macrocollinearity and microcollinearity analysis of Triticeae

**Figure 4:**
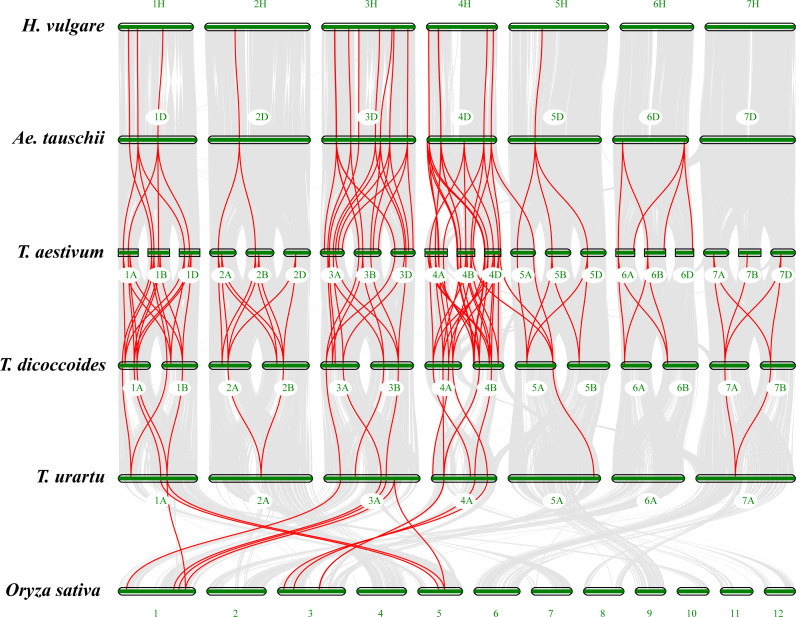
Macrocollinearity analysis of *LBD* genes between common wheat and representative plant species. Cascaded profile of macrocollinearity of different species constructed using TBtools. Selected species were ordered according to evolutionary distance with the A and D subgenomes of Chinese Spring (middle track). Collinearity relationships are shown between two adjacent species. Gray lines in the background indicate the collinear blocks within different genomes and the red lines highlight the syntenic *LBD* gene pairs.

One of the main driving forces of plant genome evolution is frequent chromosome rearrangement (Pont & Salse, 2017). The synteny analysis and phylogenetic comparison of different species can provide valuable clues for studying the evolutionary characteristics of gene families (Xie et al., 2018). In order to explore the possible phylogenetic mechanism of the *LBD* gene family in common wheat, based on the evolutionary relationship of common wheat and its related species, a macrocollinearity analysis was performed for the different species; 19, 60, 89, 22 and 14 collinear relationships were found between barley and *Ae. tauschii*, *Ae. tauschii* and common wheat, common wheat and *T. dicoccoides*, *T. dicoccoides* and *T. urartu*, and *T. urartu* and rice, respectively (Fig. 4). At the species level, compared with *B. distachyon* and rice, the uniformity of the collinear blocks among Triticeae species was higher (Fig. 4), indicating that the evolutionary relationship among Triticeae species was closer. In the *LBD* gene family, large numbers of *LBD* collinear gene pairs were produced after the two rounds of polyploidization (Dubcovsky & Dvorak, 2007), and this resulted in one *LBD* gene of diploid wheat having two homologous genes in tetraploid wheat, and each *LBD* gene of tetraploid wheat had three homologous genes in hexaploid wheat. However, the collinear relationships in different species was not in accordance with the above deduction (Fig. 4), possibly because the phylogenetic history of wheat A, B and D lineages is strongly influenced by ancestral subdivision, and the existing *LBDs* may have experienced different types of structural variation, including gene loss and copy-number variations (Cheng et al., 2018; Jiang et al., 2020). A few *LBDs* from common wheat produced gene pairs across genomes and homologous groups. For example, *TaLBD5A-5* and *TaLBD5A-6* of class II a were located in the fifth homologous group; however, the gene pairs of the two *LBD* genes from B (*TaLBD4B-10*) and D (*TaLBD4D-10*) subgenomes of common wheat were located in the fourth homologous group (Fig. 4). Consequently, we analyzed the microcollinearity of individual *LBD* genes. When *TaLBD4B-1* was used as a query gene, the results showed that its neighboring genes were relatively conserved across investigated genomes, and homologs of *TaLBD4B-1* were found in the collinearity regions of *T. urartu*, *Ae. tauschii* and subgenomes A and B of *T. dicoccoides* (Fig. S2A ) . This indicated that the *LBD* gene of common wheat subgenome D originated from *Ae. tauschii*, and the *LBDs* of common wheat subgenomes A and B derived from tetraploid durum wheat. The microcollinearity relationship of most *LBDs* was similar to the above; however, some microcollinearity of *LBDs* differed.

The microcollinearity analysis of *TaLBD4B-10* of class II a showed that its neighboring genes were relatively conserved across investigated genomes, and homologs of *TaLBD4B-10* were found in the collinearity regions of *T. urartu*, *Ae. tauschii* and subgenomes A and D of common wheat, however, the collinearity region of *T. urartu* and subgenome A of common wheat were in chromosome 5A, not chromosome 4A (Fig. S2B). Furthermore, the best matched genes of *TaLBD5A-5* and *TaLBD5A-6* were *TuLBD5A-3* and *TuLBD5A-4*, respectively. Gene *AetLBD4D-9* from *Ae. tauschii* had a “1-to-many” pairwise homology with *TaLBD4D-10* and *TaLBD4D-11* in common wheat (Fig. S2B). The evolutionary origin of these homologs may be that the ancestors of *TaLBD5A-5* and *TaLBD5A-6* were from chromosome 4A of the ancient diploid common ancestor of *Triticum–Aegilops*, and then brought to chromosome 5A along with the ancient translocation events. Gene *AetLBD4D-9* in *Ae. tauschii* was transferred to common wheat and due to gene replication, formed *TaLBD4D-10* and *TaLBD4D-11*. This also supports the presence of a translocation event of chromosomes 4A and 5A during the evolution of the ancient diploid ancestor (Chen et al., 2020b).

**Figure 5:**
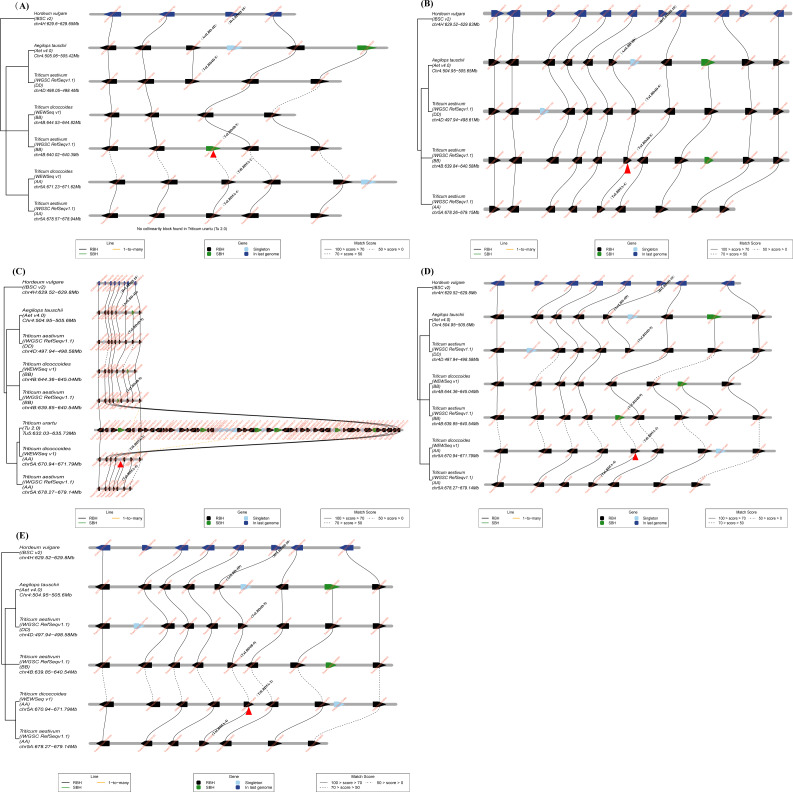
Microcollinearity analysis by TGT to track the evolutionary history of *TaLBD4B-9* gene homologs. (A–B) *TaLBD4B-9* used as query gene. The microcollinearity relationship showed that the neighboring genes of *TaLBD4B-9* were conserved across investigated genomes. However, no homolog of *TaLBD4B-9* was found in the collinearity region in subgenome B of *T. dicoccoides.* The red arrow indicates *TaLBD4B-9* (A)*.* In the microcollinearity relationship analysis the genome of *T. dicoccoides* was deleted. The neighboring genes of *TaLBD4B-9* were conserved across investigated genomes and homologs of *TaLBD4B-9* were found in all investigated genomes (B). The red arrow indicates *TaLBD4B-9*. (C–E) *Td5A-2* used as query gene. The microcollinearity relationship showed that the neighboring genes of *Td5A-2* were conserved across investigated genomes, except in the genome of *T. urartu*. A homolog of *Td5A-2* was not found in the collinearity region of *T. urartu* and many genes in this genome region of *T. urartu* did not have homologs in the genome of Chinese Spring. The red arrow indicates *Td5A-2* (C)*.* In the microcollinearity relationship analysis, the genome of *T. urartu* was deleted. The neighboring genes of *Td5A-2* were conserved across all investigated genomes. However, homologs of *TaLBD4B-9* were not found in the collinearity region of subgenome B of *T. dicoccoides.* The red arrow indicates *Td5A-2* (D)*.* In the microcollinearity relationship analysis of *Td5A-2*, the genome of *T. urartu* and subgenome B of *T. dicoccoides* were deleted. The neighboring genes of *Td5A-2* were conserved across investigated genomes and homologs of *Td5A-2* were found in all investigated genomes (E). The red arrow indicates *Td5A-2*. Black line, 1-to-1-mutual-best. Green line, 1-to-its-best. Yellow line, 1-to-many. Abbreviations: RBH, “reciprocal best hits”; SBH “single-side best hits”.

In class I d, *TaLBD5A-4*, *TaLBD4D-9* and *TaLBD4B-9* were gene pairs; using *TaLBD4B-9* as a query gene showed no collinearity block in *T. urartu*, but its neighboring genes were relatively conserved across other investigated genomes (Fig. 5A). However, homologs of *TaLBD4B-9* were not found in the microcollinearity region of subgenome B of *T. dicoccoides* (Fig. 5A). In addition, the genome of *T. dicoccoides*  was deleted from microcollinearity analysis, and homologs of *TaLBD4B-9* with high similarity were found in the microcollinearity regions of different genomes (Fig. 5B). The homolog of *TdLBD5A-2* in the microcollinearity region of the wheat A subgenome was *TaLBD5A-4*, while many genes in this genome region of *T. urartu* had no homologs in the genomes of Chinese Spring and *T. dicoccoides* (Fig. 5C). Homologs of *TdLBD5A-2* were not found in the microcollinearity regions of *T. urartu* (Fig. 5C) and subgenome B of *T. dicoccoides* (Fig. 5D). The microcollinearity relationship was further analyzed by removing the genome of *T. urartu* and subgenome B of *T. dicoccoides* (Fig. 5E), and the result showed *TdLBD5A-2* homologs of high similarity in the microcollinearity regions of different genomes. The homolog of *TaLBD4D-9* in this region of *Ae. tauschii* was *AetLBD-4D* (Figs. 5A–5E), but no such homolog was found in subgenome B of *T. dicoccoides* (Fig. 5D), suggesting that the corresponding *LBDs* in *T. urartu* and subgenome B of *T. dicoccoides* may have been lost during evolution of *T. urartu* and *T. dicoccoides* , and that *TaLBD4D-9* originated from *AetLBD-4D* and *TaLBD4B-9* was formed by duplication during the evolution of common wheat. The results suggest that this is the case, these *TaLBD* homologs are a combined result of genome translocation, polyploidization, deletion and duplication events.

### Functional diversification analysis

DIVERGE v3.0 software was used to analyze the functional divergence of *LBD* genes in different subgroups (Table 3). The results showed that the *θ* value of Type-I ranged within 0.045693–0.953022, indicating that there was an obvious difference in the degree of functional divergence in different subgroups of the *LBD* gene family. The corresponding standard error (SE) was around 0.21, and the *P*-values of classes I b/I c, I b/I d, I c/I e, I d/I a, I d/I e, I d/II a and II b/II a were higher than 0.01 (Table 3), indicating no significant difference in the evolution rate among these subgroups. The results of the other groups showed significant differences among the subgroups, suggesting significant Type-I functional differentiation. It is noteworthy that the Type-I functional divergence of *LBDs* between classes II a and I, and between classes II b and I were almost existed. The Type-II analysis showed that *θ* ranged within 0.003427–0.695788, and SE fluctuated around 0.15 (Table 3). There was significant Type-II functional divergence in classes I b/II a, I c/II a, I d/II a, II a/I a, II b/I a, I b/II b, I c/II b and I d /II b, meaning that there were some amino acid specific sites among the above subgroups, moreover all the subgroups were related to the *LBD*s in class II. Type-I analysis showed no difference between classes I d and II a, but there was evolutionary divergence between classes I d and II b; Type-II analysis showed evolutionary divergence between classes I d and II a and between classes I d and II b. The above results suggest that gene functional divergence of classes I d and II likely mainly came from the change of some critical amino acid sites, rather than a change of evolution rate.

**Table 3:** The result of Type-I and -II functional divergence.

Subgroup	I			II		
	MFE Theta	MFE se	I:P	Theta-II	Theta SE	II:P
I b/ I a	0.3791	0.1373	0.0011^**^	0.0616	0.1665	0.7117
I b/ I c	0.0457	0.1527	0.7609	0.0616	0.1436	0.6679
I b/ I d	0.0844	0.2109	0.6945	0.0450	0.1498	0.7638
I b/ I e	0.5009	0.1690	0.0007^**^	0.0591	0.2496	1.1870
I b/ II a	0.4739	0.1919	0.0062^**^	0.4601	0.1272	0.0003^**^
I b/ II b	0.4968	0.1667	0.0006^**^	0.4508	0.1200	0.0002^**^
I c/ I a	0.4327	0.1817	0.0080^**^	0.0211	0.1389	0.8791
I c/ I d	0.0843	0.2895	0.7688	0.1604	0.1136	0.1579
I c/ I e	0.4279	0.2081	0.0258	0.1399	0.2403	0.5604
I c/ II a	0.7024	0.2484	0.0023^**^	0.6111	0.0882	0.0000^**^
I c/ II b	0.7399	0.2190	0.0002^**^	0.5687	0.0879	0.0000^**^
I d/ I a	0.4050	0.2417	0.0759	0.2420	0.1325	0.0678
I d/ I e	0.1356	0.2595	0.5943	0.0485	0.2471	0.8444
I d/ II a	0.7929	0.3232	0.0105	0.6958	0.0780	0.0000^**^
I d/ II b	0.9475	0.2861	0.0005^**^	0.6040	0.0865	0.0000^**^
I e/ I a	0.6521	0.1734	0.0000^**^	0.0557	0.2319	0.8101
II a/ I a	0.5788	0.1933	0.0008^**^	0.5551	0.1141	0.0000^**^
II a/ I e	0.5558	0.2184	0.0055^**^	0.3726	0.1984	0.0604
II b/ I a	0.5890	0.1684	0.0000^**^	0.5347	0.1097	0.0000^**^
II b/ I e	0.9530	0.2012	0.0000^**^	0.3692	0.1782	0.0383
II b/ II a	0.2541	0.1977	0.1735	0.0034	0.1098	0.9751

**Notes.**

MFE, model-free method.

** Significance at *p*-values less than 0.01.

### Expression pattern of TaLBDs

**Figure 6:**
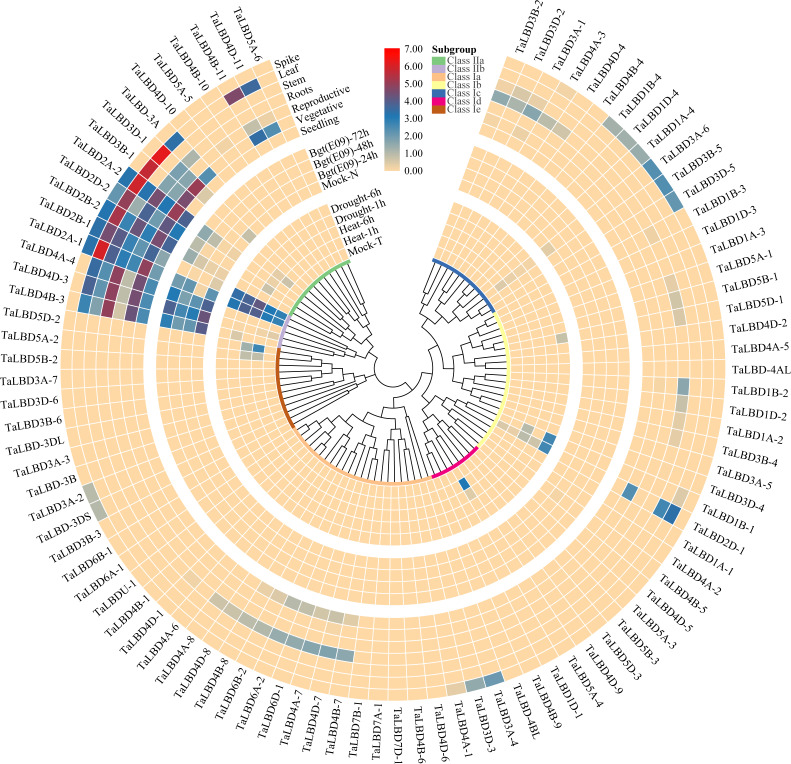
Heat map of the expression profiling of wheat *LBD* genes in different tissues, different developmental stages and under various stresses. The color scale bar represents the expression values of the genes. The phylogenetic tree was constructed using the neighbor-joining method with 1000 bootstrap replicates by MEGA X. Abbreviations: *Bgt*, *Blumeria graminis* f*.* sp*. tritici*. Mock-N, Disease-resistant wheat varieties N9134 without the infection of *Bgt*, Mock-T, Heat-resistant wheat cultivar TAM107 without heat or drought treatment at seedling stage.

**Figure 7:**
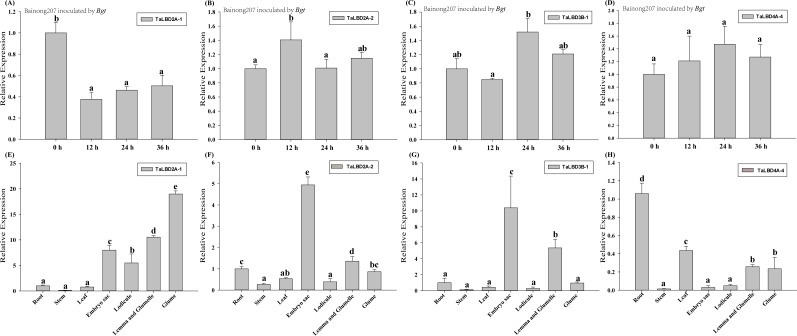
Relative expressions of four *TaLBD* genes after *Bgt* inoculation and in different tissues by qRT-PCR. (A–D) Expression profiling of four *TaLBD* genes in response to *Bgt*. (E–G) Tissue-specific expression pattern of four *TaLBD* genes in common wheat Bainong207. Data were normalized to *TaTubulin* gene. The values are the means of three technical replicates of one biological experiment. Error bars indicate the standard error. Different letters indicate significant differences assessed using Duncan’s honestly significant difference test (*P* < 0.05). It is not significant when the letters of each treatment were the same, and significant only when the letters were completely different. The details of the primer sequences were listed in Table S1. Abbreviations: *Bgt*, *Blumeria graminis* f*.* sp*. tritici*.

The expression patterns of gene family members are helpful to predict their potential biological functions (Zhao et al., 2019). In order to elucidate the potential role of *TaLBDs*, their expression patterns were studied by qRT-PCR or in silico expression profiling. Expression patterns of *TaLBD* s in different tissues (roots, stems, leaves of seedling stage and spikes at flowering stage), different developmental stages (seedling, vegetative and reproductive stages), under two abiotic stresses (drought and heat) and one biotic stress (powdery mildew pathogen E09) were analyzed using the wheat RNA-seq data from public databases (Borrill, Ramirez-Gonzalez & Uauy, 2016; Pearce et al., 2015). The expression level was measured as tags per million (TPM), and we assumed that expression was high if TPM ≥ 2.5; moderate if 2.5 >TPM ≥ 1.5; low if 1.5 >TPM >0; and undetectable if TPM = 0, as reported by Zhao et al. (2019) . Cluster analysis showed diverse expression patterns in *LBD*s of common wheat (Fig. 6). Tissue expression analysis showed that 14 genes (15.05%) were highly expressed in at least one of the four tissues, 36 genes (38.71%) were expressed in at least one tissue and 43 genes (46.24%) were not detected in the tissues. Most of the genes in class I were not responsive to heat, drought and powdery mildew stresses, and their expression levels in the different developmental stages and different tissues were low or undetectable. In contrast, some genes in class II were responsive to stress and their expression were high in different tissues and developmental stages (Figs. 6–7, Fig. S3). The expression levels of *TaLBD4B-11*, *TaLBD4D-11*, *TaLBD-3A*, *TaLBD3B-1*, *TaLBD3D-1*, *TaLBD2A-2*, *TaLBD2B-2* and *TaLBD2D-2* in class II a were higher in the vegetative than other developmental stages, higher in leaves than other tissues, and expressions of *TaLBD3B-1* and *TaLBD3D-1* in spikes and stems were undetectable. Furthermore, upon *Bgt* infection, *TaLBD2A-2*, *TaLBD2B-2* and *TaLBD2D-2* expression was upregulated at 72 h (Fig. 6). The expression of *TaLBD4D-10*, *TaLBD5A-5*, *TaLBD4B-10* and *TaLBD5A-6* in class II was not detected in different developmental stages, different tissues or under different stress (Fig. 6). In class II b, expression of *TaLBD4B-3*, *TaLBD4D-3* and *TaLBD4A-4* were responsive to *Bgt* inoculation. Moreover their expression levels were downregulated after *Bgt* inoculation, were undetectable in spikes but high in other tissues (Fig. 6). The expression levels of *TaLBD2A-1* and *TaLBD2B-1* increased significantly under heat and drought stresses, and were high in reproductive stage and different tissues (except in roots) (Fig. 6). Among all the analyzed genes, only *TaLBD2A-1* and *TaLBD2B-1* were highly expressed in the analyzed above ground tissues and in response to all three stresses. Conserved roles were observed for all three homologous genes from different genomes, such as *TaLBD2A-2*, *TaLBD2B-2*, *TaLBD2D-2* and *TaLBD4B-11*, *TaLBD4D-11*, *TaLBD-3A*. Gene expression was generally in agreement with the expected subfamily-specific expression pattern, suggesting broad conservation of function of *LBD*s during wheat evolution.

The expression patterns of the four *TaLBD* s (*TaLBD2A-1*, *TaLBD4A-4*, *TaLBD2A-2* and *TaLBD3B-1*) that were expressed in most of all analyzed tissues were further analyzed by qRT-PCR analysis in different tissues such as roots, stems, leaves, embryo sacs, lodicules, mixed tissues of lemma and glumelle, and glumes and following powdery mildew stress. After *Bgt* inoculation, *TaLBD2A-1* expression was downregulated at 12, 24 and 36 h (Fig. 7A) and the expression trend was similar to RNA-seq data obtained from the expVIP database. Expression of *TaLBD2A-2* was upregulated at 12 h and returned to the original level at 24 h (Fig. 7B); The relative expression level of *TaLBD3B-1* at 24 h was about 1.58 times of 12 h and returned to the original level at 36 h (Fig. 7C); and *TaLBD4A-4* was not response to *Bgt* (Fig. 7D). The results suggested that *TaLBD2A-1*, *TaLBD2A-2* and *TaLBD3B-1* may play an important role in wheat response to biotic stress. The expression patterns of *TaLBD4A-4*, *TaLBD2A-2* and *TaLBD3B-1* under *Bgt* stress were different to RNA-seq data obtained from the wheat expVIP database, possibly because the expression patterns in plant–pathogen compatible interactions differ to incompatible interactions. In different tissues, the expression levels of *TaLBD2A-1*, *TaLBD2A-2* and *TaLBD3B-1* were higher in embryo sacs, glumes, glumelles and lemmas, and lower in stems (Figs. 7E–7F), suggesting that the above three genes regulate reproductive growth and are involved in embryo sac development in common wheat. The expression level of *TaLBD4A-4* was high in roots and low in stems (Fig. 7G). The expression trends of the four analyzed genes in roots, stems and leaves were similar to RNA-seq data, indicating that it is reasonable and feasible to use RNA-seq data to evaluate the transcriptional expression level of wheat genes.

## Discussion

### Evolutionary relationship of LBD genes in common wheat and related species

Gene duplication generates functional divergence, which is essential for environmental adaptability and speciation (Hittinger & Carroll, 2007). In the process of evolution, duplicated gene pairs can experience functional divergence, leading to sub-functionalization, non-functionalization or neo-functionalization of genes (Prince & Pickett, 2002). Gene duplication is one of the major mechanisms for gene family expansion (Cannon et al., 2004). *Triticum aestivum* has experienced two rounds of complex polyploidization in the evolutionary process, and the A subgenome of common hexaploid wheat evolved from *T. urartu*, and the D subgenome evolved from *Ae. tauschii* (Chen et al., 2020b). The phylogenetic history of wheat lines of A, B and D lineage are strongly influenced by ancestral subdivision (Jiang et al., 2020). Our results showed that *T. aestivum* (hexaploid), *T. dicoccoides* (tetraploid), *H. vulgare* (diploid), *Ae. tauschii* (diploid) and *T. urartu* (diploid) contained 94 (IWGSC:88; TGAC:6), 49, 31, 27 and 27 *LBD* genes, respectively, with a ratio about 9:5:3:3:3 (Table 1), which was basically consistent with the distribution of subgenome multiples. Changes of the gene dosage may result in changes of the stoichiometry protein complexes, which may in turn have phenotypic effects (Birchler et al., 2005). Some researchers believed that, compared with traditional terms “paralog” and “ortholog,” “homolog” can more accurately and reasonably explain the relationship between new polyploid plant genes (Chen et al., 2020b). Based on this viewpoint, we speculate on the main evolutionary process of *LBDs* in hexaploid wheat according to the information of homologs, classification and structure of *LBDs*. In *T. aestivum*, 50 pairs of homologous *LBDs* were found, and 15 pairs were found in *T. dicoccoides*.

Gene duplication analysis indicated that there was no tandem duplication in common wheat *LBDs*, thus, tandem repeats had no significant effect on *LBD* family evolution in common wheat, and a similar view was also verified in other species (Chen et al., 2020b; Lu et al., 2018). Most *LBD* gene pairs in common wheat belonged to same homologous group (Fig. 2); most of them evolved from their ancestral species during natural polyploidization, which is the traditional evolution of most *LBD* genes in *T. aestivum*. Some *LBD*s evolved in different ways: *TaLBD5A-5* and *TaLBD5A-6* of class II a were on chromosome 5A, while the *LBDs* which were their gene pairs were all on the fourth homologous group. The motifs of TaLBD4B-11 and TaLBD4D-11 were the same, they were highly similar to TaLBD5A-6; TaLBD4B-10 and TaLBD5A-5 were also the same, they were highly similar to TaLBD4D-10 (Fig. 3). Furthermore, both the macrocollinearity (Fig. 4) and the gene microcollinearity (Fig. S2B) showed that *TaLBD5A-5* and *TaLBD5A-6* may have originated from chromosome 4A of the diploid ancestor, and the fragment carrying *LBD*s translocated to chromosome 5A during the formation of the diploid progenitor of the A genome and further evolved into *T. dicoccoides* and *T. aestivum* (Chen et al., 2020b; Jorgensen et al., 2017). In this research, *TaLBD5A-4*, *TaLBD4D-9* and *TaLBD4B-9* were gene pairs; the motifs of TaLBD4B-9, TaLBD4D-9 and TaLBD5A-4 were highly similar (Fig. 3), *TaLBD5A-4* and *TdLBD5A-2* had high similarity but no corresponding homologs in the microcollinearity region of *T. urartu*; and *TaLBD4D-9* and *AetLBD-4D* had high similarity, but no corresponding homologs in the microcollinearity region of *T. dicoccoides* subgenome B (Figs. 5A–5E). This suggests that the corresponding *LBDs* in *T. urartu* and subgenome B of *T. dicoccoides* may have been lost during evolution of *T. urartu* and *T. dicoccoides*, *TaLBD4D-9* originated from *AetLBD-4D*, and *TaLBD4B-9* may have been formed by duplication during the evolution of common wheat (Chen et al., 2020b; Golicz, Batley & Edwards, 2016). The *TaLBD* homologs were likely a combined result of chromosome translocation, polyploidization, deletion and duplication events.

Type-I differentiation represents the difference in gene evolution rate, and Type-II differentiation represents changes in the physicochemical properties of amino acids (Yang et al., 2020). The functional divergence of common wheat *LBD* gene subgroups differed (Table 3). The difference between the two large class of *LBD*s lies in the non-conservative protein structure (Lu et al., 2018), classes I and II were classified according to their specific protein sequences of the LOB domain in the N-terminus (Shuai, Reynaga-Pena & Springer, 2002), suggesting that the second large class of *LBD*s represented by classes II a and II b was functionally differentiated compared with most *LBD*s in class I partly due to the sequences of the LOB domain in the N-terminus. In Type-I differentiation, class I d showed no significant difference from class II a, but showed significant difference from class II b, possibly because some *LBD*s in classes I d and II a were involved in chromosomal translocation events and affected the Type-I *θ*-value between classes I d and II a (Chen et al., 2020b; Dvorak et al., 2018; Jorgensen et al., 2017).

Therefore, we speculate that *LBDs* in *T. aestivum* had three main different evolutionary patterns. Firstly, most of the *LBDs* in the three subgenomes of *T. aestivum* came from their respective ancestral species; during this period, there was no significant change in the gene structure, no obvious gene replication and the characteristics of *LBD*s of their ancestors were retained. Secondly, some *LBD* gene rearrangements were caused by translocation events of chromosomes 4A and 5A before the formation of the diploid progenitor of the A genome, and the genes evolved into *T. dicoccoides* and hexaploid wheat. Thirdly, some *LBD*s were lost during formation of tetraploid wheat. The incomplete *LBD* gene of *Ae. tauschii* evolved into hexaploid wheat through polyploidization and the *LBDs* with complete LOB domains evolved from the ancestral gene.

### Function differentiation of the *TaLBD* gene family in common wheat

The *LBD* gene family encode plant-specific transcription factors, which have been shown to play an important role in various aspects of plant growth and development (Iwakawa et al., 2002; Majer & Hochholdinger, 2011; Shuai, Reynaga-Pena & Springer, 2002; Yu et al., 2020a; Yu et al., 2020b). They are therefore promising targets for crop breeding and improvement. It has been reported that *Crl1* is essential for crown root and lateral root formation in rice (Inukai et al., 2005). Genes *AtLBD16*, *AtLBD18* and *AtLBD29* have proven to be involved in lateral root initiation (Goh et al., 2019; Lee et al., 2019a). Cluster analysis showed diverse expression patterns of *LBD*s in common wheat. Tissue expression analysis showed that most *LBD*s in class II b had high expressions in roots, but most genes in class I were low or undetectable. In roots, *TaLBD4B-3*, *TaLBD4D-3* and *TaLBD4A-4* in class II b had high expression compared to stem and leaf (Fig. 6), and this result was verified by qRT-PCR (Fig. 7H). Among the four verified genes, *TaLBD4A-4* expression was higher in roots than other tissues, suggesting that this gene was involved in root development. A maize ortholog of LOB, *RA2*, has been shown to regulate reproductive growth and is involved in the morphogenesis of maize inflorescence (Bortiri et al., 2006; Vollbrecht et al., 2005). The indeterminate *gametophyte1* (*ig1*) gene of maize belongs to the *LBD* gene family and is required for embryo sac and leaf development (Evans, 2007) . In the present study, expression levels of *TaLBD2A-1*, *TaLBD2A-2* and *TaLBD3B-1* were higher in embryo sacs, lemma and glumelle and lower in stems (Figs. 7E–7G), suggesting that these genes were related to embryo sac development in common wheat. In potato, expressions of *StLBD2-6* and *StLBD3-5* were induced under drought stress (Liu et al., 2019); in sorghum, *SbLBD32* is highly induced under various stresses (Wang et al., 2010). Gene *AtLBD20* is a *F. oxysporum* susceptibility gene (Thatcher et al., 2012b); and *CsLOB1* is a citrus bacterial canker susceptibility gene  in *Citrus sinensis* (Hu et al., 2014; Zhang et al., 2017). Thus, it is reasonable to predict that the *LBDs* may play roles in plant stress resistance. In the present study, some of the genes in class II were expressed in different tissues, developmental stages and were responsive to stress (Fig. 6, Fig. S3). These results are consistent with the previous report that the *LBDs* belonging to the smaller class II of LBD proteins were responsive to multiple pathogens and abiotic stresses, suggesting functions of *LBDs* in plant defense responses (Thatcher, Kazan & Manners, 2012a).

## Conclusions

In the present study, a total of 228 *LBD* members were identified from Triticeae species, and phylogenetic relationship analysis of *LBD* members classified them into two main classes and seven subgroups. Macro- and micro-scale collinearity analyses of Triticeae species suggested that some *LBD* genes from wheat produced gene pairs across subgenomes of chromosomes 4A and 5A and that the complex evolutionary history of *TaLBD4B-9* homologs was a combined result of chromosome translocation, polyploidization, gene loss and duplication events. The expression analysis revealed that some *TaLBDs* in class II responded to powdery mildew, regulated reproductive growth and were involved in embryo sac development in common wheat. This study elucidated the evolution and diversity of *LBD* genes in Triticeae species, and their potential roles in plant growth and stress response, which provided a foundation for the functional studies of these genes in the future.

##  Supplemental Information

10.7717/peerj.11811/supp-1 Supplemental Information 1 Phylogenetic relationships, gene structures and conserved protein motifs of *LBD* genes in Triticeae species (A–D) Phylogenetic tree, protein motifs and gene structures of Triticeae species *LBD* s grouped into classes I a, I b, I c and I e, respectively. (A) The phylogenetic tree was constructed using the neighbor-joining method with 1000 bootstrap replicates by MEGA X. (B) The motif composition of LBD proteins. The motif compositions were analyzed by the online tool MEME, different motifs for LBD proteins are indicated by different colored boxes and numbered 1–20. (C) Exon–intron structure of *LBDs*. Gene structure analysis of *LBD* genes was performed using TBtools.

10.7717/peerj.11811/supp-2 Supplemental Information 2 Microcollinearity analysis by TGT to track the evolutionary history of *TaLBD4B-1* and *TaLBD4B-10* gene homologs (A) Microcollinearity analysis among different species helped reveal the gene-specific evolutionary history. The microcollinearity relationship showed that neighboring genes of *TaLBD4B-1* were relatively conserved across investigated genomes, and homologs of *TaLBD4B-1* were found in the collinearity regions in *T. urartu*, *Ae. tauschii* and subgenomes A and B of *T. dicoccoides.* The red arrow indicates *TaLBD4B-1*. (B) The microcollinearity relationship showed that the neighboring genes of *TaLBD4B-10* were conserved across investigated genomes, and homologs of *TaLBD4B-10* were found in the collinearity region in *T. urartu* and *Ae. tauschii* and subgenomes A and D of common wheat; however, the collinearity region of *T. urartu* and subgenome A of common wheat were in chromosome 5A, not in chromosome 4A. Black line, 1-to-1-mutual-best. The red arrow indicates *TaLBD4B-10.* Green line, 1-to-its-best. Yellow line, 1-to-many. Abbreviations: RBH,“reciprocal best hits”; SBH “single-side best hits”.

10.7717/peerj.11811/supp-3 Supplemental Information 3 Relative expressions of 2 *TaLBD* genes after NaCl treatment by qRT-PCR

10.7717/peerj.11811/supp-4 Supplemental Information 4 Primers used for qRT-PCR

10.7717/peerj.11811/supp-5 Supplemental Information 5 Characteristics of LBD genes in eight species

10.7717/peerj.11811/supp-6 Supplemental Information 6 Raw data for Fig. 7

10.7717/peerj.11811/supp-7 Supplemental Information 7 Raw data for Fig. S3

10.7717/peerj.11811/supp-8 Supplemental Information 8 Protein sequences of various species

10.7717/peerj.11811/supp-9 Supplemental Information 9 Raw data for the new LOB HMM model generated

10.7717/peerj.11811/supp-10 Supplemental Information 10 Raw data for the macro-collinearity analysis

## Additional Information and Declarations

### Data Availability

The following information was supplied regarding data availability: The raw measurements are available in the Supplemental Files.
