# Supplementary figures and images for: Genome-wide identification and characterization of the Lateral Organ Boundaries Domain (LBD) gene family in polyploid wheat and related species
Source: PeerJ. 2021 Aug 11;9:e11811. doi: 10.7717/peerj.11811 (PMC8364319; doi:10.7717/peerj.11811)

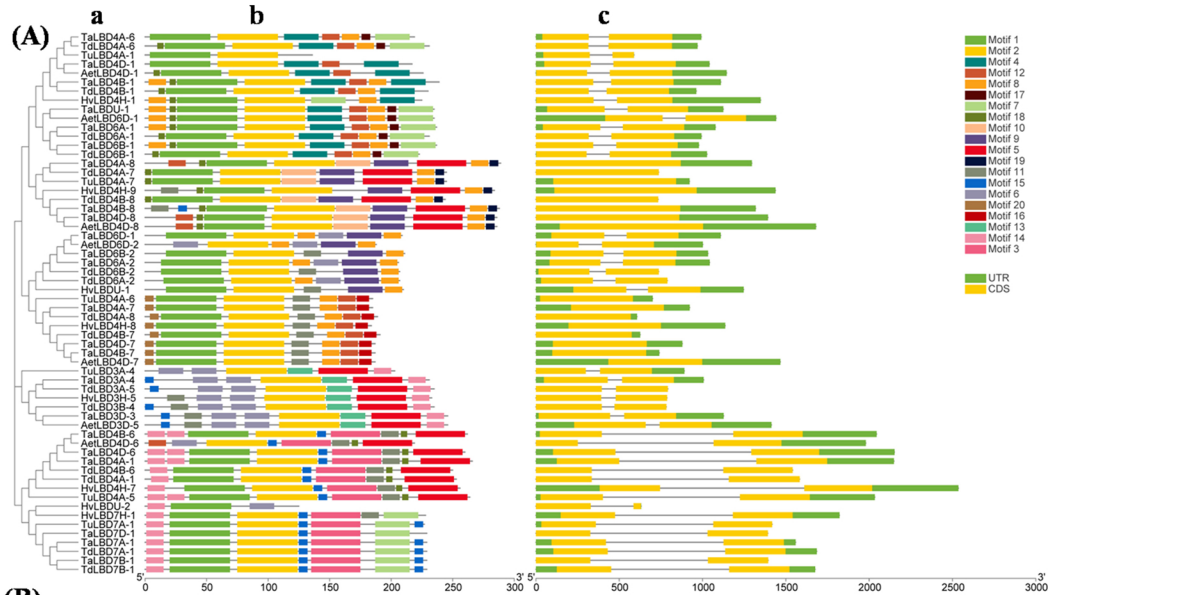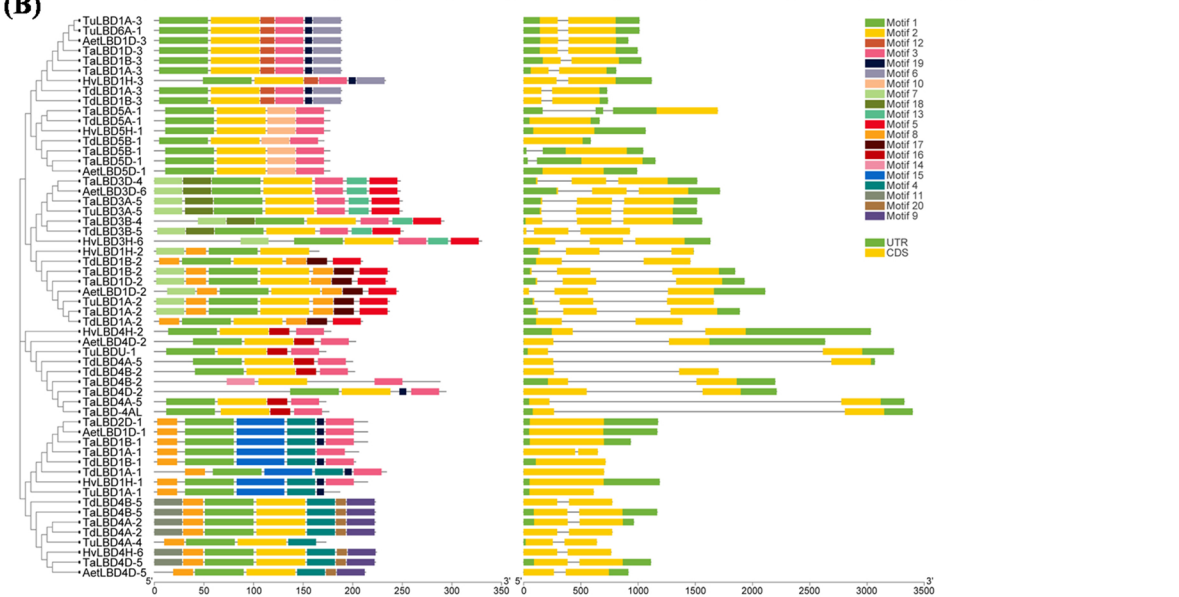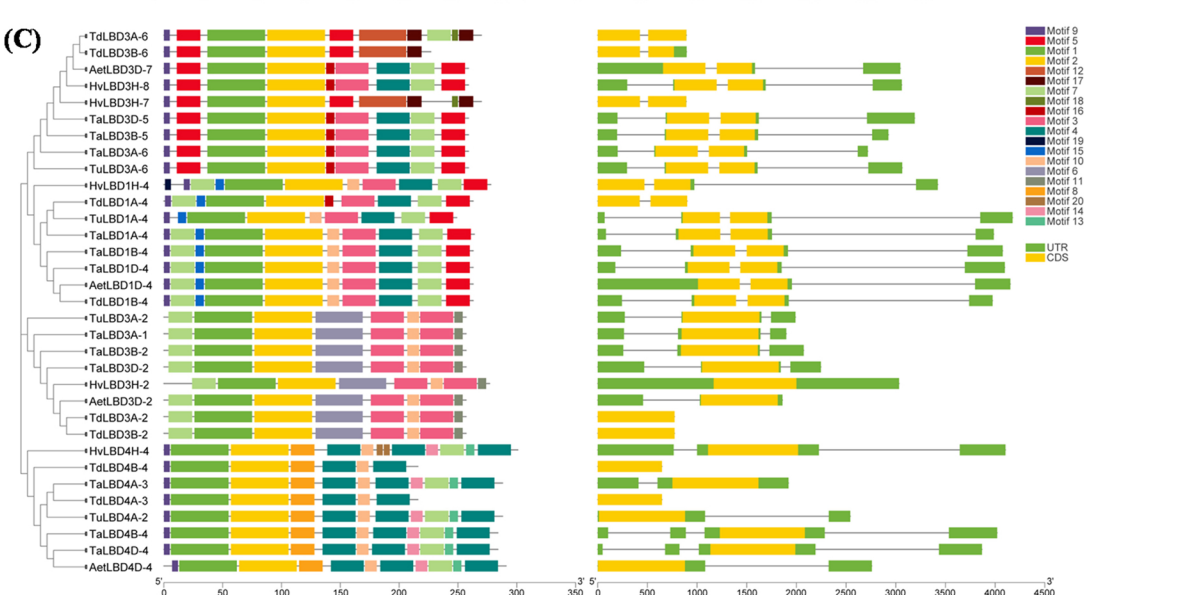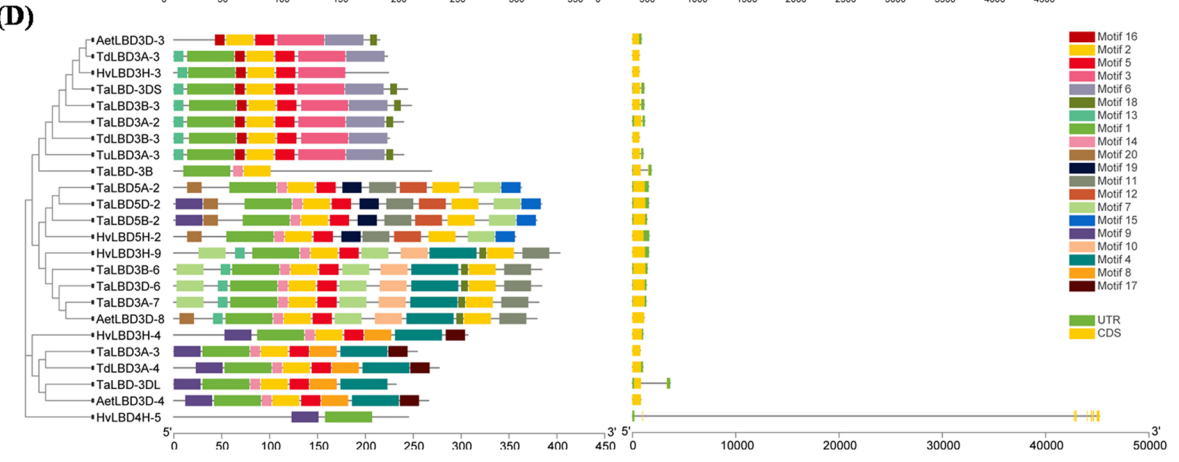

Supplement: Supplemental Information 1 — (A–D) Phylogenetic tree, protein motifs and gene structures of Triticeae species LBD s grouped into classes I a, I b, I c and I e, respectively. (A) The phylogenetic tree was constructed using the neighbor-joining method with 1000 bootstrap replicates by MEGA X. (B) The motif composition of LBD proteins. The motif compositions were analyzed by the online tool MEME, different motifs for LBD proteins are indicated by different colored boxes and numbered 1–20. (C) Exon–intron structure of LBDs. Gene structure analysis of LBD genes was performed using TBtools. [file peerj-09-11811-s001.pdf]

**(A)**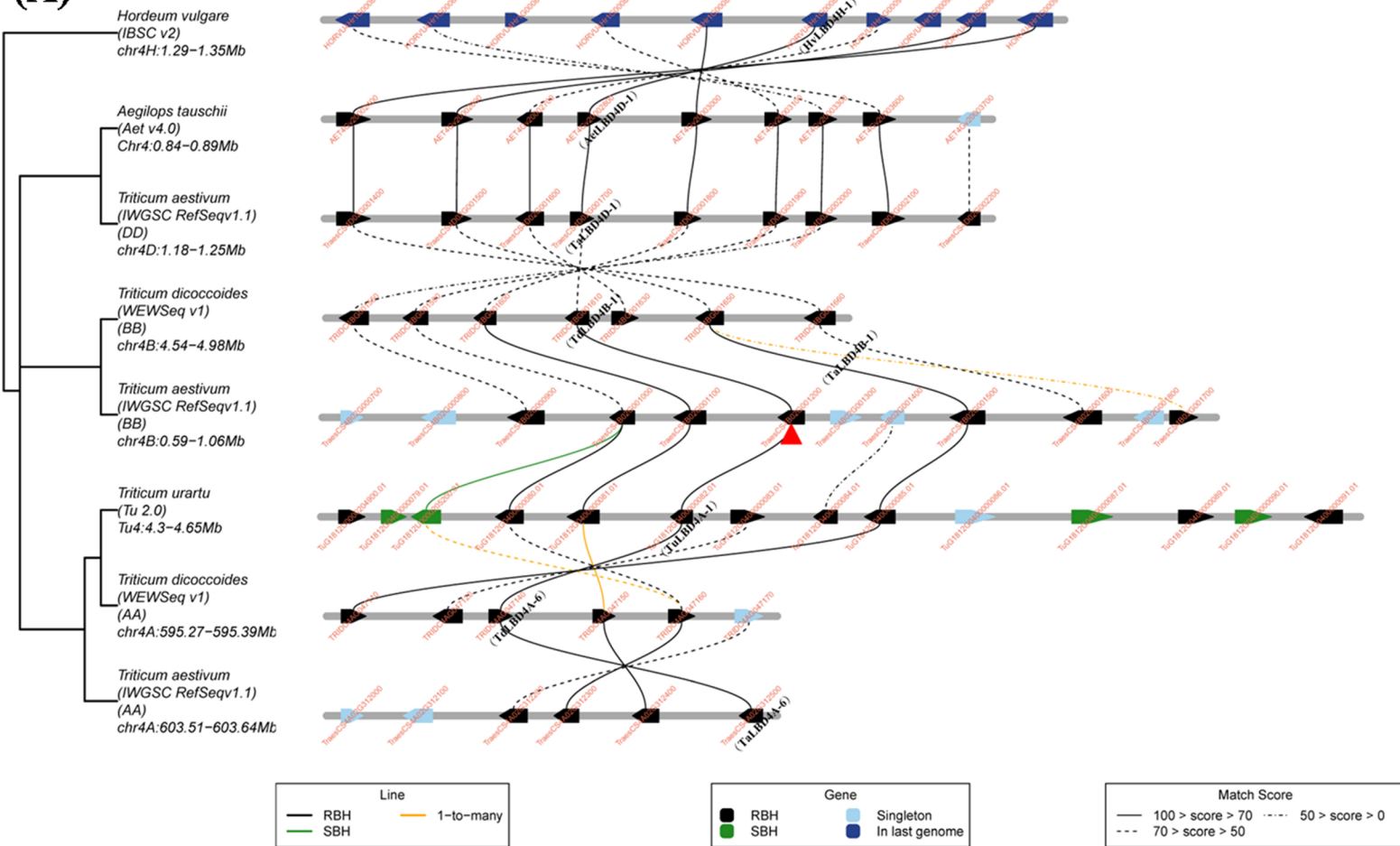**(B)**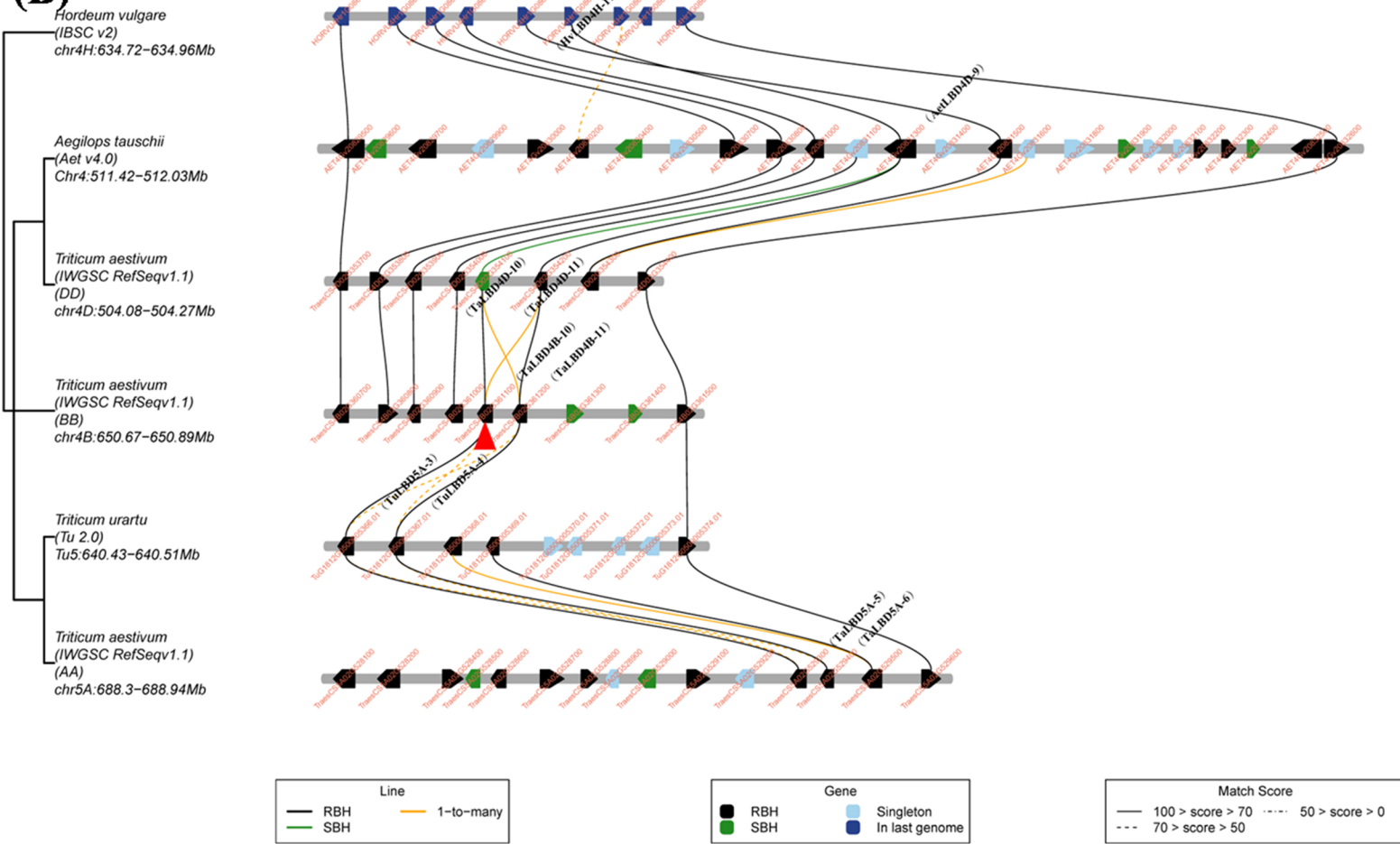

Supplement: Supplemental Information 2 — (A) Microcollinearity analysis among different species helped reveal the gene-specific evolutionary history. The microcollinearity relationship showed that neighboring genes of TaLBD4B-1 were relatively conserved across investigated genomes, and homologs of TaLBD4B-1 were found in the collinearity regions in T. urartu, Ae. tauschii and subgenomes A and B of T. dicoccoides. The red arrow indicates TaLBD4B-1. (B) The microcollinearity relationship showed that the neighboring genes of TaLBD4B-10 were conserved across investigated genomes, and homologs of TaLBD4B-10 were found in the collinearity region in T. urartu and Ae. tauschii and subgenomes A and D of common wheat; however, the collinearity region of T. urartu and subgenome A of common wheat were in chromosome 5A, not in chromosome 4A. Black line, 1-to-1-mutual-best. The red arrow indicates TaLBD4B-10. Green line, 1-to-its-best. Yellow line, 1-to-many. Abbreviations: RBH,“reciprocal best hits”; SBH “single-side best hits”. [file peerj-09-11811-s002.pdf]

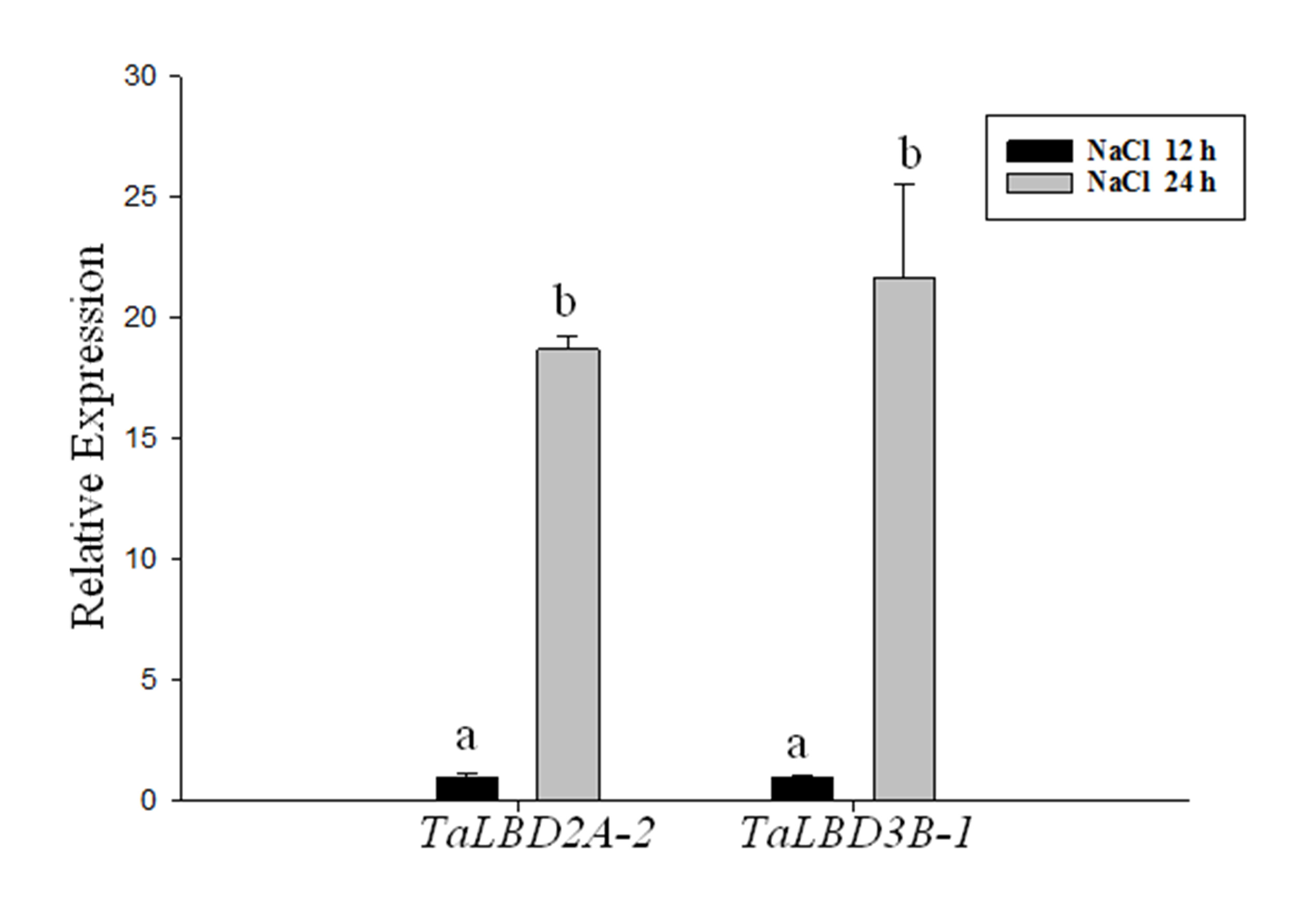

Supplement: Supplemental Information 3 [file peerj-09-11811-s003.jpg]
