# Supplementary material for: Genome-wide identification and characterization of the Lateral Organ Boundaries Domain (LBD) gene family in polyploid wheat and related species
Source: PeerJ. 2021 Aug 11;9:e11811. doi: 10.7717/peerj.11811 (PMC8364319; doi:10.7717/peerj.11811)
Supplement: Supplemental Information 4 [file peerj-09-11811-s004.docx]

**Table S1. Primers used for qRT-PCR**

| Name | Sequence |
| --- | --- |
| TaLBD2A-1F | 5’- GTCAACGTCGGGGATGAG |
| TaLBD2A-1R | 5’- TACAGCAGCGACTGGAACAC |
| TaLBD2A-2F | 5’- CTTCCTCGCCAAGTTCTACG |
| TaLBD2A-2R | 5’- CAGTTGTTGGACCAGAGCAG |
| TaLBD3B-1F | 5’- CCAAGTCCAAGAGCTTCTCC |
| TaLBD3B-1R | 5’- CCCTCGGACGACTCTTCTTC |
| TaLBD4A-4F | 5’- TCTTCGTCGCCAAGTTCTTC |
| TaLBD4A-4R | 5’- AGTTGCTGGTCCACATGAGC |
